# Control of structure and spin texture in the van der Waals layered magnet CrSBr

**DOI:** 10.1038/s41467-022-32737-8

**Published:** 2022-09-15

**Authors:** J. Klein, T. Pham, J. D. Thomsen, J. B. Curtis, T. Denneulin, M. Lorke, M. Florian, A. Steinhoff, R. A. Wiscons, J. Luxa, Z. Sofer, F. Jahnke, P. Narang, F. M. Ross

**Affiliations:** 1 Department of Materials Science and Engineering, Massachusetts Institute of Technology, Cambridge, MA 02139 USA; 2 John A. Paulson School of Engineering and Applied Sciences, Harvard University, Cambridge, MA USA; 3 Department of Physics, Harvard University, Cambridge, MA USA; 4 Ernst Ruska-Centre for Microscopy and Spectroscopy with Electrons and Peter Grünberg Institute, Forschungszentrum Jülich, 52425 Jülich, Germany; 5 Institut für Theoretische Physik, Universität Bremen, P.O. Box 330 440, 28334 Bremen, Germany; 6 Department of Electrical Engineering and Computer Science, University of Michigan, Ann Arbor, MI USA; 7 Department of Chemistry, Columbia University, New York, 10027 NY USA; 8 Department of Inorganic Chemistry, University of Chemistry and Technology Prague, Technická 5, 166 28 Prague 6, Czech Republic

**Keywords:** Materials for devices, Magnetic properties and materials

## Abstract

Controlling magnetism at nanometer length scales is essential for realizing high-performance spintronic, magneto-electric and topological devices and creating on-demand spin Hamiltonians probing fundamental concepts in physics. Van der Waals (vdW)-bonded layered magnets offer exceptional opportunities for such spin texture engineering. Here, we demonstrate nanoscale structural control in the layered magnet CrSBr with the potential to create spin patterns without the environmental sensitivity that has hindered such manipulations in other vdW magnets. We drive a local phase transformation using an electron beam that moves atoms and exchanges bond directions, effectively creating regions that have vertical vdW layers embedded within the initial horizontally vdW bonded exfoliated flakes. We calculate that the newly formed two-dimensional structure is ferromagnetically ordered in-plane with an energy gap in the visible spectrum, and weak antiferromagnetism between the planes, suggesting possibilities for creating spin textures and quantum magnetic phases.

## Abstract (web summary)

Van der Waals magnetic materials are composed of atomically thin magnetically ordered layers stacked together. Here, aiming to control magnetism locally, Klein et al use an electron beam to create small regions where van der Waals layers are orientated perpendicular to the rest of the sample.

## Introduction

The observation of long-range magnetic order in the van der Waals (vdW) magnets CrI_3_^1^, Cr_2_Ge_2_Te_6_^2^, and Fe_3_GeTe_2_^3^ has expanded material platforms for the study of low-dimensional magnetism^4^. Such pioneering work has strong relevance for exploring fundamental questions in physics^5–7^, including probing the Mermin–Wagner–Hohenberg theorem^8,9^ and realizing spintronic and magneto-electric devices^10–18^ that allow voltage-controlled magnetic and spin-related properties^3,19–21^. Microscopically, such properties are governed by local coupling between spins that are strictly connected to the underlying crystal lattice^22,23^. Thus, creating new degrees of freedom requires tailoring local structural properties^24^. Beyond the influence of the periodic crystal structure on the local magnetic coupling, chemical and structural defects, as well as interfaces between domains, twins or confined geometries, have been shown to produce exotic low-dimensional spin textures with novel chiralities^25–28^, but controlled engineering of such defect topologies has proven to be challenging for further advancement in this field^29^.

In contrast to 3D materials, layered magnets offer superior design opportunities for engineering both periodic and non-periodic structures and, therefore, spin textures down to a single atomic level. Intrinsic (naturally occurring) spin textures below the critical temperature are made up of domain walls formed due to thermodynamically driven spin neutrality, while twin/grain boundaries and multicrystals are structural modulations that generally form naturally in structures to increase the entropy of the system. A hitherto overlooked aspect of vdW magnets is their potential for atomic scale structural modifications using electron or ion beam irradiation for the controlled creation of zero- to high dimensional defect topologies that range from single vacancies to locally induced crystal phase changes^30^. Of particular interest is the exploration of interfaces and their proximity effects that provide a playground for studying local chirality^25^, magnetic anisotropy, and spin canting on ultra-short length scales. Deterministic engineering of previously studied vdW magnets has remained elusive due to their poor structural stability, since full encapsulation and protection in a glovebox environment and sophisticated fabrication methods are required ^1,3^.

In this report, we demonstrate a versatile approach to engineer the structural landscape with the potential to modulate the spin textures in layered vdW magnets with a high degree of spatial control. We conduct our study on the recently rediscovered vdW layered magnet CrSBr^31–34^ that constitutes an ideal candidate due to its known amenability for intrinsic structural changes^32^ and its highly promising electronic, optical, and magnetic properties^31–37^. CrSBr is an air-stable ferromagnetic (FM) insulator in the monolayer limit with a direct band gap of ~1.6 eV^33–35^ hosting tightly bound magneto-excitons^37^. The magnetic easy axis is in-plane with an antiferromagnetic (AFM) interlayer coupling in bulk with a Néel temperature of *T*_*N*_ = 132 K^32,36^ and a recently suggested intermediate soft magnetic phase up to 160 K^35,36^. The combination of these properties and the crystal stability makes CrSBr ideal for nanoscale structural modification, and hence the creation of spin textures potentially even with atomic resolution, a key requirement for building advanced spintronic and magneto-electric devices.

## Results

### Local crystal phase change

**Fig. 1:**
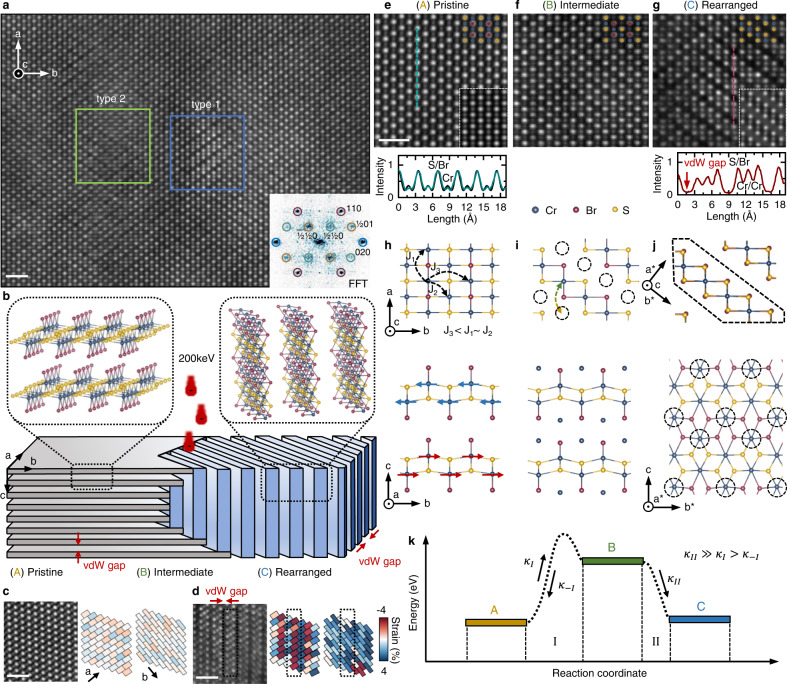
Electron beam-induced local crystal phase change in CrSBr. **a** Low magnification STEM-HAADF image of a multilayer CrSBr flake imaged for 100*s* acquisition time with a beam energy of 200 keV at a beam current of 60 pA corresponding to an electron dose of ~130 pm^−2^ (see Methods). Inset: Corresponding FFT showing double periodicity peaks (cyan and brown circles). The scale bar is 1 nm. **b** Schematic illustration of the electron beam-induced structural rearrangement of CrSBr from (A) pristine with out-of-plane stacking through (B) intermediate phase to (C) the rearranged structure exhibiting in-plane stacking. **c** STEM-HAADF image of pristine multilayer CrSBr and corresponding strain maps along the *a*- and *b*-direction, obtained from S/Br atom column positions (see Methods). **d** STEM-HAADF image of the rearranged crystal structure (C) and corresponding strain maps. **e** High-magnification STEM-HAADF image of multilayer CrSBr. Bright and medium intensity spots correspond to S/Br and Cr atom columns, respectively. Experimental and simulated line profiles show the S/Br and the Cr columns. Inset: Multislice simulated STEM image of a pristine CrSBr sample (see Methods). The scale bar is 1 nm. **f** STEM-HAADF image of multilayer CrSBr in the intermediate state (B). **g** A representative image of the rearranged CrSBr. Experimental and simulated line profiles show the migration of Cr. Inset: Corresponding multislice simulation STEM image of the rearranged structure. The scale bar is 1 nm. **h** Top-view of the crystal structure of CrSBr and exchange couplings J_1_, J_2_, and J_3_ between next nearest neighbor Cr atoms and side-view of the bilayer with the AFM ordering. **i** Top- and side-view of the intermediate crystal structure (B). **j** Top- and side-view of the DFT relaxed crystal structure of rearranged CrSBr. **k** Reaction diagram of the electron beam-induced structural rearrangement.

We first demonstrate the electron beam-induced structural rearrangement. Fig. 1a shows a scanning transmission electron microscopy high-angle annular dark-field (STEM-HAADF) image of a typical multilayer CrSBr crystal area after electron beam irradiation (See also Supplementary Figs. 1, 2, 3). We observe two different types of new structures: type 1 (Supplementary Video 1) is the newly formed in-plane stacked 2D material, while type 2 (Supplementary Video 2) represents a layer stacking fault that forms due to a buildup of strain and potential local loss of Br (discussed in detail in Supplementary Figs. 8, 9, 15). We focus on structure type 1 throughout the remainder of this report.

The local beam-induced structural rearrangement of the CrSBr crystal is illustrated in Fig. 1b. Dosing pristine CrSBr (A) with 200 keV electrons results in the reversible migration of Cr atoms into the vdW gap to form a metastable intermediate crystal state (B) that eventually nucleates into a new magnetic (see below) 2D material (C) that is stacked along the in-plane direction. This striking rearrangement opens a vdW gap in the *a*-*b*-plane. While for pristine CrSBr, we find lattice distances of *a* = 350.59 pm and *b* = 477.03 pm, the vdW gap of the new structure is clearly visible from changes in lattice distances in the *a*- and *b*-direction. This is quantified in strain maps of pristine (Fig. 1c) and fully arranged CrSBr (Fig. 1d) obtained from S/Br atom column positions. Generally, even pristine CrSBr crystals exhibit a large variation of up to ±2% over wide areas, likely due to the layered nature of the material (see Supplementary Figs. 5, 7, 9 for detailed analysis of lattice distances and strain). A similar elongation along the *b*-direction is also observed in Li-intercalated FeOCl^38,39^.

High-magnification STEM-HAADF images of the three different states (A, B, and C) are shown in Fig. 1e–g, along with their corresponding top- and side-view crystal structure models in Fig. 1h–j. To our advantage, in pristine CrSBr, columns containing S/Br and Cr atoms are spatially well separated in a top-view perspective along the *c*-direction and can be easily distinguished by their image contrast difference, in excellent agreement with multislice simulations (see inset Fig. 1e). The intensity variation of the atomic columns is also visible in the line profile in Fig. 1g yielding a contrast of $\frac{{I}_{Cr}}{{I}_{S/Br}} \sim 80\%$. Irradiating with electrons creates a local and intermediate crystal phase (Fig. 1f) visible from local intensity variations of the Cr atom column intensities, which over a total of 80 s of exposure spontaneously rearranges into the new in-plane stacked 2D material (Fig. 1g).

The electron beam-driven mechanism of this structural rearrangement is described in a reaction coordinate diagram in Fig. 1k. Local irradiation disrupts the bonding structure of Cr atoms, overcoming the activation energy such that the Cr can migrate into the vdW gap with a rate *κ*_*I*_. This state is the local metastable intermediate state (B) (see Fig. 1f, j). From this state, Cr can either reverse to its original position with a rate *κ*_−*I*_ or irreversibly relax in the stable final rearranged state (C) at a rate *κ*_*II*_. The top- and side-view of the newly formed 2D material is shown in Fig. 1j. Cr atoms from every second diagonal have migrated into a proximal diagonal.

From the side-view along the a* direction of a single new 2D layer (highlighted in Fig. 1j), it is apparent that the migrated Cr atoms (see dashed circles) are bonded to four Br and two S atoms instead of the initial two Br atoms and four S atoms. The new crystal symmetry is similar to a 1*T* phase of CrBr_2_^40^. The experimental observation is in excellent agreement with the multislice simulated STEM image from the ab initio relaxed structure model. The corresponding calculated change in image contrast can also directly be observed in the line cuts along the *a*-direction showing the net loss and surplus of Cr atoms in alternating Cr atom columns, clear evidence for the rearrangement and emergence of the new 2D material. Moreover, the change in the *a*- and *b*-directions is also in excellent agreement between experiment (Fig. 1c, d) and our relaxed ab initio DFT crystallographic model. We find that the ratio between experimental (theoretical) *a*- and *b*-dimensions increases from 1.3596 (1.3553) to 1.3946 (1.3834).

### Nucleation kinetics

**Fig. 2:**
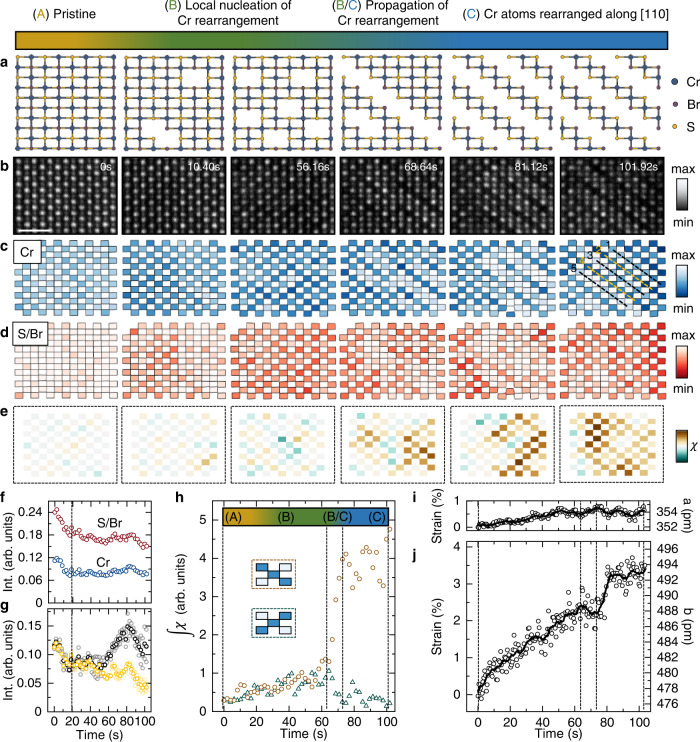
Nucleation and growth kinetics of new structural phases in CrSBr driven by electron beam irradiation. **a** Schematic illustration (top-view) of the electron beam-induced structural rearrangement of Cr atoms. **b** STEM-HAADF images of the CrSBr at 0, 10.40, 56.16, 68.64, 81.12, and 101.92 s. The flake is imaged with a beam energy of 200 keV at a beam current of 75 pA, corresponding to an electron dose of ~160 pm^−2^. The scale bar is 1 nm. Voronoi diagrams of **c** Cr and **d** S/Br from a Voronoi tesselation. **e** Nucleation maps (degree of local nucleation, *χ*) derived from the intensity difference of next nearest Voronoi cells along the two different Cr diagonals. **f** Time evolution of the average column intensity of Cr (blue circles) and S/Br (red circles). **g** Time evolution of averaged intensities from Cr diagonals on (black circles) and off (orange circles) the rearranged layer. See highlighted dashed lines in **c**. **h** Time evolution of the integrated degree of nucleation. Inset: Schematic of the two different orientations along the diagonals. The pristine CrSBr (A) shows local nucleation and reversible Cr migration (B) until ~62 s, where a sudden global ordering occurs (B/C), resulting in the rearrangement and relaxation into new 2D layers. **i**, **j** Time evolution of the net strain along the *a*- and the *b*-direction. The strain relaxation in the *b*-direction at ~62 s is accompanied by global crystal rearrangement. Scale bars are 1 nm.

We are particularly motivated by the possibility of forming these structural modifications within a matrix of the naturally grown structure because we can then utilize the spin structures of both structural domains to engineer low-dimensional chiral and/or topological spin textures at their interface. In order to analyze the time evolution of the process described in Fig. 1k more quantitatively, we collect a sequence of 200 STEM-HAADF images at a frame rate of 1.92 frames/s (total of 101.92 s) and electron energy of 200 keV with a dose of 160 pm^−2^. The complete time evolution of the lattice rearrangement is shown in Fig. 2a.

Selected STEM-HAADF images for 0, 10.40, 56.16, 68.64, 81.12, and 101.92 s are shown in Fig. 2b. Since the atom column intensity (image contrast) follows the dependence *I* ∝ *Z* ∗ *N* (*Z* is the atomic number of each element), we can track column intensities to directly infer changes in atom column stochiometry as a function of time. Column intensity maps obtained from a Voronoi tesselation of Cr and S/Br (Voronoi diagrams) are shown in Fig. 2c and d (see Supplementary Figs. 4, 10 for details on the analysis).

While both the Cr Voronoi diagram and the real space STEM-HAADF image reflect the time evolution of the rearranged structure, the S/Br atom columns do not exhibit such a redistribution of intensity over this time frame. Importantly, as the crystal undergoes rearrangement, there is no large change in overall stochiometry (see Fig. 2f), but Cr atoms from diagonals (see dashed black lines 1, 3, and 5 in Fig. 2c at 101.92 s) migrate to proximal diagonals (orange dashed lines 2 and 4). The migration becomes even more apparent by tracking the average intensity of the two different Cr diagonals, as shown in Fig. 2g. For *t* > 60 s, a splitting of the intensities occurs, a direct consequence of the electron beam-induced Cr migration. The striking migration of Cr in CrSBr is also further demonstrated by the filling of patterned 1D Cr vacancy line defects under irradiation (see Supplementary Fig. 16 and Supplementary Videos 3, 4).

To study the nucleation kinetics and crystal rearrangement in space and time, we analyze Cr Voronoi cell intensities from Fig. 2c using the next nearest neighbor Cr atom columns along the two different diagonals (see inset Fig. 2h and Supplementary Fig. 11 for details). This analysis is sensitive to both local and global changes in the CrSBr nucleation kinetics. The spatial distribution of nucleation (*χ*) at different times is shown in the corresponding nucleation maps (Fig. 2e). Moreover, we derive and summarize the full-time evolution from the integrated value of the nucleation maps (degree of nucleation) as shown in Fig. 2h, which represents a precise measure of the degree of rearrangement along one diagonal or the other.

At 0 s the CrSBr is pristine (A) with a homogeneous distribution of Cr atom column intensities. However, for *t* > 0 s, the degree of nucleation monotonically increases, reflecting local stochastic nucleation events of the Cr rearrangement (metastable state B) that is driven by the electron beam irradiation. Such events spatially appear as high values in the nucleation maps. For *t* > 60 s, a sudden global Cr rearrangement propagates through the analyzed area and the crystal fully rearranges into its final state (C). The full global rearrangement occurs on short time-scales (~10 s). We observe the same trend in reciprocal space (see Supplementary Fig. 12). Moreover, aligning the scan direction (e.g., along the [110] direction) manifests in an additional degree of freedom to control the preferred rearrangement direction (see Supplementary Fig. 3).

To further understand what microscopically triggers the sudden rearrangement, we quantify the time evolution of the net strain in the crystal along the *a*- and *b*-directions (see Fig. 2i and further details on the analysis in Supplementary Fig. 13). For the *a*-direction, the overall increase is less than 1%, which is due to the compensation of small and large strain values from the alternating lattice distortion within each newly formed 2D layer and the opening of the vdW gap (see Fig. 1d). However, in the *b*-direction for 0 < *t* < 60 s, we observe a continuous buildup of net strain that is accompanied by an increase in local nucleation (Cr migration events). A striking observation is that the net strain decreases simultaneously with the global rearrangement into the new 2D material. We interpret this as the lattice reaching a critical strain value due to the increased number of local Cr migration events that, in order to minimize its free energy, fully relax into the rearranged lattice structure (C) to release its accumulated strain.

We note that the phase transformation readily occurs at the acceleration voltage of 200 kV but does not show up at 60 kV even for extended scan times of 5 min. This is likely due to the beam energy being well below the displacement threshold energy of the Cr atom. Moreover, we varied the beam current at 200 kV between 60–105 pA to explore the effect of electron dose on the phase transformation. We typically observe the phase transformation (line defect formation) after 80 s at 75 pA beam current, as shown in the data in Fig. 2 and Supplementary Video 1. For higher beam currents, the line defects form more quickly (within 50−60 s e.g., for 105 pA, see Supplementary Video 5) due to the higher rate of electron-induced structure transformation, resulting in an enhanced rate from state A to state B and eventually to state C. The phase transformation is followed by a material loss that occurs for extended scan times (rapid vacancy formation and eventually holes in the material). However, it is noted that the structure can be frozen out in the rearranged state by modulating the beam current to minimize further beam damage. In general, the phase transformation is reproducible over several samples with different layer thicknesses, and the transformation is robust and still observable after exposing to ambient conditions over an extended period of time. Additionally, we find that the line defects can be created locally and individually when the beam is scanned along a line rather than over a larger area (Fig. 4).

### Electronic structure and magnetic interactions

**Fig. 3:**
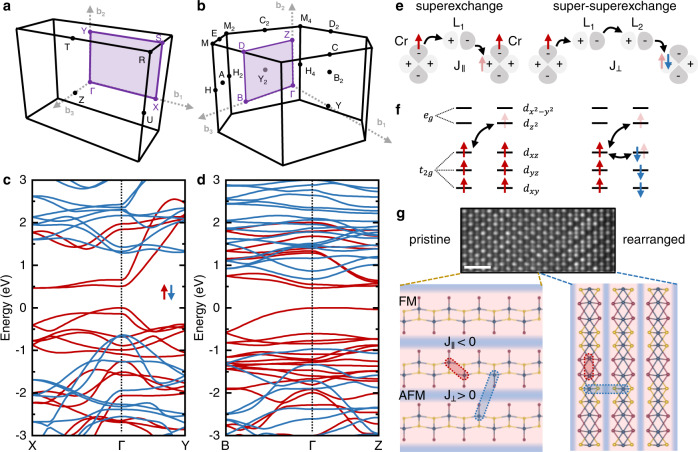
Electronic and magnetic properties of rearranged CrSBr. **a** Brillouin zone of bulk **a** pristine and **b** rearranged CrSBr and their corresponding spin-resolved DFT electronic band structure **c**, **d**. **e** FM intralayer superexchange and AFM interlayer super-superexchange between the Cr atoms mediated by one or two ligands. **f** Level scheme of intralayer superexchange interaction between Cr atoms. Crystal-field splitting into doubly degenerate *e*_*g*_ and triple degenerate *t*_2*g*_ orbitals and the corresponding hopping terms for FM and AFM coupling. **g** Pristine CrSBr exhibits intralayer FM order and interlayer AFM order for *T* < *T*_*N*_. The rearranged CrSBr shows intralayer FM order and weak AFM order. The scale bar is 1 nm.

To elucidate the electronic and magnetic properties of the rearranged structure, we calculate the electronic band structure and determine the magnetic interactions from a simplified tight-binding model of the relevant orbitals. Figure 3a, b show the full Brillouin zone of the pristine and rearranged CrSBr. The corresponding DFT-calculated spin-polarized band structures for the essential high symmetry points are shown in Fig. 3c, d. The lowest energy bands of the rearranged CrSBr are fully spin-polarized with a direct gap transition at the Γ point and with energy comparable to the pristine CrSBr, suggesting an FM ground state. Since we are interested in the magnetic ordering of the rearranged CrSBr and in the spin texture the local rearrangement creates, we further determine the intralayer and interlayer exchange interaction for the pristine and the rearranged CrSBr. In this calculation, we determine the intralayer exchange interaction *J*_∥_ using superexchange mediated by the ligand, and the interlayer interaction *J*_⊥_ by super-superexchange via two ligands (see Fig. 3e). By performing strong-coupling perturbation theory (see Fig. 3e, f) using the effective hoppings and crystal-field splittings extracted from spin-unpolarized ab initio band structure calculations, we indeed find the in-plane superexchange interaction is ferromagnetic, with coordination and unit-cell averaged superexchange of order of *J*_∥_ = −13 meV for typical correlation parameters (here a minus sign indicates ferromagnetism).

We also compute the interlayer magnetic interactions via the super-superexchange mechanism. Owing to the large separation between layers and roughly linear bond-angle, we find that the interlayer coupling is weakly antiferromagnetic, with a typical value being of order *J*_⊥_ = 1.5 meV. We therefore expect that for a relatively large range of temperatures, the system exhibits quasi-two-dimensional ferromagnetism, which is locally well-formed within the planes but only weakly coupled between the planes, creating unusual spin textures in interfaces between rearranged and pristine CrSBr (see Fig. 3g).

Note that, given the quasi-two-dimensional nature of the rearranged structure, it is also important to determine the magnetocrystalline anisotropies, which arise from the spin–orbit corrections to the superexchange. We can roughly estimate their size^41,42^ as follows. Bromine has the largest atomic spin-orbit coupling, with *ξ*_Br_ ~220 meV^41^. This enters into the superexchange correction via second-order perturbation theory with suppression by *ξ*_Br_/Δ_Br_ ~ 0.06, implying that the anisotropic exchange interactions should be of order *ξ*_Br_/Δ_Br_*J*_∥_ ~0.8 meV, which is comparable to the interlayer exchange *J*_⊥_. We therefore expect that the true form of the magnetic ordering will be determined by a combination of the interlayer antiferromagnetism and in-plane anisotropies, and a more thorough analysis would be the subject of future calculations. Future measurements with high spatial resolution (e.g., differential phase contrast (DPC) STEM^27^, NV-magnetometry^43^) will help to further explore the magnetic structure down to the atomic scale.

### Opportunities for writing spin textures on-demand

**Fig. 4:**
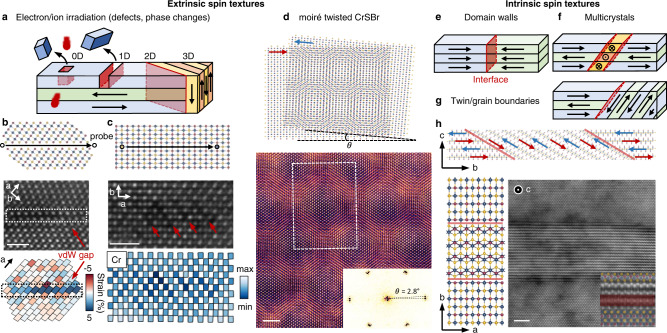
The opportunity for spatial control of spin textures. **a** Design of extrinsic spin textures by electron or ion irradiation via deterministic manipulation of crystal defects and phases. **b** A representative example showing the creation of a single Cr line vacancy by monitoring the scanning direction of the 200 keV electron probe (along a pre-selected Cr line), introducing an artificial vdW gap as shown in the STEM-HAADF image and the corresponding local strain map. The scale bar is 1 nm. **c** Scanning along the *b*-direction creates a zig-zag pattern due to selective migration of Cr atoms into the proximal vdW gap and local rearrangement. The scale bar is 1 nm. **d** CrSBr moiré superlattice with controlled twist angles (~2.8^∘^ in this instance) imaged at 200 keV. The scale bar is 2 nm. Intrinsic spin textures that naturally occur are **e** domain walls, **f** twin or grain boundaries and **g** multicrystals. **h** Intrinsic spin texture induced by twinning in a four-layer CrSBr imaged at 200 keV. The twin boundary is tilted by an angle of ~30.9° with respect to the *c*-axis and creates an atomically defined interface. Scale bar is 2 nm.

The above calculations show that complex spin textures can be obtained by inserting, through targeted irradiation, regions of the new structure into a CrSBr matrix. The structural amenability of CrSBr provides a wide range of possibilities for programming spin textures into the lattice with a range of different topologies (0–3D) (see Fig. 4a). To emphasize this point, we also demonstrate the flexibility of CrSBr for writing additional atom-level structural modifications. Fig. 4b shows a representative example of monitoring the scanning direction of the electron probe to selectively create a single Cr line vacancy, which artificially introduces a vertical vdW gap (see Fig. 4b) within the horizontally vdW-bonded matrix. The absence of Cr atoms in the STEM-HAADF image and the formation of a vdW gap from the local strain analysis suggest the feasibility of modifying the crystal structure, and hence magnetic properties, on-demand at the atomic scale. Scanning the electron probe across both S-Br and Cr atom columns creates a zig-zag pattern due to the selective migration of Cr atoms when the probe is on a Cr site. This is further shown by the variation in the analysed Cr atom column intensity. Another example of an extrinsic spin texture is the intentional introduction of a twist between two CrSBr sheets to create a superlattice (see Fig. 4d). Twisted magnets are of particular interest for creating exotic quantum magnetic phases^44^. Beyond the intentionally created textures are also intrinsic spin textures that can naturally occur and by themselves represent exciting magnetic systems given the layered nature of the host material. Examples include domain walls, multicrystals, and twin and grain boundaries (see Fig. 4e–g). Indeed, we observe examples of twin boundaries with a few nm width within exfoliated bulk crystals (see Fig. 4h and Supplementary Fig. 14), creating atomically defined interfaces with different magnetic anisotropy on either side.

## Discussion

We believe that these results lay the groundwork for creating and studying new structural phases and related spin textures in vdW magnets, ultimately even down to the atomic scale. Channel thicknesses of locally rearranged magnetic layers are shorter than state-of-the-art silicon transistor gate lengths, opening up exciting avenues for realizing new spintronic device designs combining out-of-plane and in-plane stacked 2D materials. The development of a means to modify magnetic structure locally may be harnessed to generate topological non-trivial spin textures with an unprecedented degree of control over dimensionality. Moreover, the intricate interplay of magnetization and localization effects of excitons will be interesting for future investigation, potentially leading to engineering spin-Hamiltonians in situ.

Furthermore, the atomistic study of Cr migration sheds further light on ionic movement^45,46^, intercalation^47,48^, and therefore phase transformations in van der Waals materials that can be expanded and more generally applied to other materials with similar crystal symmetry like CrSeBr, CrSBr_*x*_Cl_1−*x*_, CrOCl, CrOBr, TiOCl, VOCl, or FeOCl to create new magnetic phases ^33,34,49–51^.

The degree of structural control demonstrated here will also be critical in obtaining a mechanistic understanding of proximity-induced effects in low-dimensional heterostructures. Finally, magnetic and spin-orbit degrees of freedom in 2D magnets allow for flexible interaction engineering as well as simultaneous control of electronic band structure and topology, essential towards realizing universal classes of quantum many-body Hamiltonians and using vdW heterostructures as programmable quantum simulation platforms.

## Methods

### Crystal synthesis

CrSBr crystals were prepared by a direct reaction from for elements. Chromium (99.99%, -60 mesh, Chemsavers, USA), bromine (99.9999%, Sigma-Aldrich, Czech Republic), and sulfur (99.9999%, Stanford Materials, USA) were mixed in a stochiometric ratio in a quartz ampoule (35 × 220 mm) corresponding to 15 g of CrSBr. Bromine excess of 0.5 g was used to enhance vapor transport. The material was pre-reacted in an ampoule using a crucible furnace at 700 °C for 12 h, while the second end of the ampoule was kept below 250 °C. The heating procedure was repeated two times until the liquid bromine disappeared. The ampoule was placed in a horizontal two-zone furnace for crystal growth. First, the growth zone was heated to 900 °C, while the source zone was heated at 700 °C for 25 h. For the growth, the thermal gradient was reversed and the source zone was heated from 900 to 940 °C and the growth zone from 850 to 800 °C over a period of 7 days. The crystals with dimensions up to 5 × 20 mm were removed from the ampule in an Ar glovebox.

### Structural characterization of bulk CrSBr

We characterized grown CrSBr bulk crystals using SEM, EDS, XPS, and XRD (see Supplementary Figs. 17, 18,19). The EDS data show homogeneous distribution of Cr, S, and Br. High-resolution XPS reveals the presence of Br^−^ and S^2−^ anions and the Cr primarily in the 3+ oxidation state. The X-ray diffraction reveals a pure single-phase CrSBr with a high preferential orientation owing to the layered vdW structure.

### Sample fabrication

Bulk CrSBr flakes were exfoliated using the Scotch tape method. Individual flakes and their thickness were determined by atomic force microscopy (AFM) and optical phase contrast. Exfoliated flakes were transferred to TEM-compatible sample grids using cellulose acetate butyrate (CAB) as polymer handle^52^. The CAB was then dissolved in acetone and the TEM grids rinsed in isopropanol before critical point drying. Twisted CrSBr samples were fabricated using the dry viscoelastic transfer method.

### Optical spectroscopy

We optically characterized the CrSBr bulk crystal by Raman and photoluminescence (PL) spectroscopy at room temperature (see Supplementary Fig. 19). The PL spectrum of a multilayer CrSBr flake was measured exciting at 785 nm with an excitation power of ~50 μW. For the Raman data, we used a low-frequency filter set to obtain both Stokes and Anti-Stokes Raman modes. Monochromatic laser excitation at 532 nm was used at a power of 100 μW.

### STEM imaging

STEM imaging was performed with a probe-corrected Thermo Fisher Scientific Themis Z G3 60-200 kV S/TEM operated at 200 and 60 kV with a probe convergence semi-angle of 19 and 30 mrad, respectively. The probe size of the aberration-corrected electron beam (at 200 kV and 17 mrad convergence angle) is sub-Angstrom (0.6−1 Å). A collection semi-angle of 63–200 mrad was used for STEM-HAADF imaging. The beam current at 200 kV varied between 60–105 pA. The data were collected using Velox software (Thermo Fisher). The frame size is typically 1024 × 1024 pixel and the dwell time is 500 ns/pixel.

### STEM data analysis

In order to analyze sequences of STEM-HAADF images, movies are drift corrected before subsequent data processing. After drift correction, the position of the S/Br atom columns are determined by using atom-column indexing^53^. Since Cr atom column intensities change throughout the video due to the Cr migration, we use the positions of proximal S/Br atom columns in order to approximate the Cr atom column position. Based on each set of x-y-coordinates from both S/Br and Cr atom columns, we perform a Voronoi tesselation. Each Voronoi cell represents a single atom column. In order to obtain the atom column intensity, we integrate the intensity in the original STEM-HAADF image by masking out a polygon defined by each individual Voronoi cell. To determine local bond distances, we determine the distance between S/Br atom columns along the different crystallographic directions. Corresponding details are provided in Supplementary Figs. 4, 5, 6, 7.

### STEM multislice simulation

STEM multislice simulations were performed on DFT relaxed crystal structure of the pristine and rearranged CrSBr using the Dr. Probe software. We used simulation parameters similar to the experimental conditions, including beam energy, convergence angle, and collection angle, as well as the sample thickness. Simulated HAADF images were convolved with a Gaussian kernel with full width at half-maximum of 70 pm to approximately account for the finite size of the effective electron source.

### Ab initio calculations

The ab initio calculations were performed using density functional theory (DFT), using the Vienna ab initio simulation package (VASP), and the projected augmented wave method^54,55^. Bulk (vertical) structures were modeled with supercells containing 6 (12) atoms. The atomic and electronic structures were determined using the PBE (Perdew–Burke–Ernzerhof) functional. Van der Waals corrections were added in the Tkatchenko–Scheffler approximation^56^. A plane wave basis with an energy cutoff of *Ecut* = 500 eV and a (7 × 5 × 2) ((2 × 4 × 4)) Monkhorst-Pack-point sampling was employed for the bulk (vertical) structure. Structural relaxations were performed until the forces were smaller than 10^−3^eV Å^−1^. To generate the TB Hamiltonian used in the spin-wave theory, the Wannier90 package^57^ was employed.

### Magnetic structure

We predict the form of the magnetic structure by applying standard superexchange theory. We model the on-site energy levels, including electron-electron interactions, by a Hubbard–Kanamori + crystal-field Hamiltonian^58^, which in the rotationally invariant limit assumes the form

1 $$H=\mathop{\sum}\limits_{j}\mathop{\sum}\limits_{a}{\varepsilon }_{a}^{(j)}{\hat{n}}_{ja}+\frac{1}{2}\left(U-\frac{3}{2}{J}_{H}\right){\hat{n}}_{j}({\hat{n}}_{j}-1)-{J}_{H}{\hat{{{{{{{{\bf{S}}}}}}}}}}_{j}^{2}$$

with Hubbard-*U* modeling Coulomb repulsion, Hund’s intra-atomic exchange *J*_*H*_, which favors highest spin multiplicity, and single-particle crystal-field splitting ${\varepsilon }_{a}^{(j)}$. Here we have introduced *d*-band electronic operators ${\hat{n}}_{ja}{\hat{n}}_{j},\,{\hat{{{{{{{{\bf{S}}}}}}}}}}_{j}$ which measure the total number of *d*-electrons in crystal-field level ${\varepsilon }_{a}^{(j)}$, the total *d*-electron occupation, and total spin of the Cr ion on-site *j*, respectively. The neglect of terms due to rotational symmetry breaking is a crude approximation, but partly justified by the small crystal-field splittings. Following ref. 33, we compute the exchange for a range of values of *U* = 3.0−4.2 eV and *J*_*H*_ = 0.6–1.2 eV. We obtain the crystal-field splittings from the ab initio calculations.

We determine the superexchange energies by performing strong-coupling perturbation theory up to second order in the effective *d*−*d* hoppings. See ref. 59. for a similar treatment in the case of CrI_3_ and CrCl_3_, and also ref. 60. For a dimer of Cr ions in the *d*^3^*d*^3^ configuration, we find the superexchange interaction *J*_se_ by computing ${J}_{{{{{{{{\rm{se}}}}}}}}}=\frac{2}{9}({E}_{{{{{{\mathrm{FM}}}}}}}-{E}_{{{{{{\mathrm{AFM}}}}}}})$, which assumes that the spin-interactions are a simple *SU*(2) invariant (this is guaranteed in the absence of spin–orbit coupling) Heisenberg form, and FM and AFM indicate the states $\left|S=\frac{3}{2},\;m=\frac{3}{2}\right\rangle \otimes \left|S=\frac{3}{2},\;m=\frac{3}{2}\right\rangle$ and $\left|S=\frac{3}{2},\;m=\frac{3}{2}\right\rangle \otimes \left|S=\frac{3}{2} \;,\;m=-\frac{3}{2}\right\rangle$, respectively. In terms of the orbitally-resolved hoppings ${t}_{\beta \alpha }^{12},\;{t}_{\beta \alpha }^{21}$ from orbital *α* on site 1 to orbital *β* on site 2, and vice-versa, we find the result

2 $${J}_{{{{{{{{\rm{se}}}}}}}}}=\frac{2}{3}\mathop{\sum}\limits_{\alpha \beta }\left[\frac{1}{3}\frac{{t}_{\beta \alpha }^{12}{t}_{\beta \alpha }^{21}{n}_{\alpha }{n}_{\beta }}{U+2{J}_{H}+{\delta }_{\beta \alpha }^{(21)}}-\frac{{J}_{H}{t}_{\beta \alpha }^{12}{t}_{\beta \alpha }^{21}{n}_{\alpha }(1-{n}_{\beta })}{{(U+{\delta }_{\beta \alpha }^{(21)})}^{2}-{(2{J}_{H})}^{2}}\right]+(1\leftrightarrow 2)$$

where ${\delta }_{\beta \alpha }^{(21)}={\varepsilon }_{\beta }^{(2)}-{\varepsilon }_{\alpha }^{(1)}$ is the energy of the *d*−*d* excitation and *n*_*α*_ = 〈*n*_*jα*_〉 is the occupation of the level *α* and is taken to be 1 for the lower three levels and 0 for the upper two levels.

The final ingredient is to derive the effective ligand-mediated shopping, which we approximate by

3 $${t}_{\beta \alpha }^{12}=-\mathop{\sum}\limits_{L}{\left[{\hat{V}}^{L\to 2}\cdot {\hat{h}}_{L}^{-1}\cdot {\hat{V}}^{1\to L}\right]}_{\beta \alpha },$$

where the product is understood as a matrix product over the intermediate orbital states, and *L* sums over the participating ligands (for edge-sharing octahedra, there are two). Here, ${\hat{h}}_{L}$ is the on-site ligand-*L* block of the Wannier Hamiltonian, and ${\hat{V}}^{L\to 2},\;{\hat{V}}^{1\to L}$ are the appropriate Cr-ligand hybridizations, also obtained from wannierized ab initio calculations. We find that, except for extreme values of the Hubbard-*U* and Hund’s-*J*_*H*_, the intralayer exchanges are robustly FM.

In order to fix the interlayer ordering, we must compute the interlayer super-superexchange interactions. In this case, we again apply Eq. (1), but now using (alongside the direct *d*-band hoppings) the effective *d*-band hoppings of

4 $${t}_{\beta \alpha }^{11}=+ \mathop{\sum}\limits_{{L}_{1},{L}_{2}}{\left[{\hat{V}}^{2\leftarrow {L}_{2}}{\hat{h}}_{{L}_{2}}^{-1}{\hat{V}}^{{L}_{2}\leftarrow {L}_{1}}\cdot {\hat{h}}_{{L}_{1}}^{-1}\cdot {\hat{V}}^{{L}_{1}\leftarrow 1}\right]}_{\beta \alpha },$$

with *L*_1_ and *L*_2_ labeling all participating pairs of ligands. We find that this produces a weak AFM interlayer exchange *J*_⊥_ ~1.5 meV, as in the pristine case but now with a re-oriented magnetic structure.

## Supplementary information

Supplementary Information

Supplementary Video legends

Supplementary Video 1

Supplementary Video 2

Supplementary Video 3

Supplementary Video 4

Supplementary Video 5

## Data Availability

Data supporting the findings of this work are provided in the paper and/or the Supplementary Information. Other relevant data can be obtained from the corresponding authors upon reasonable request.

## Code availability

The codes used for data analysis as well as ab initio calculations are available from the corresponding authors on reasonable request.
