# Supplementary material for: Control of structure and spin texture in the van der Waals layered magnet CrSBr
Source: Nat Commun. 2022 Sep 15;13:5420. doi: 10.1038/s41467-022-32737-8 (PMC9478124; doi:10.1038/s41467-022-32737-8)
Supplement: Supplementary file 1 — Supplementary Information [file 41467_2022_32737_MOESM1_ESM.pdf]

Supplementary Information - Control of structure and spin texture in the van der Waals layered magnet CrSBr

J. Klein,[1,*] T. Pham,[1] J. D. Thomsen,[1] J. B. Curtis,[2,3] T. Denneulin,[4] M. Lorke,[5] M. Florian,[5] A. Steinhoff,[5] R. A. Wiscons,[6] J. Luxa,[7] Z. Sofer,[7] F. Jahnke,[5] P. Narang,[2,†] and F. M. Ross[1,‡]

[1]*Department of Materials Science and Engineering, Massachusetts Institute of Technology, Cambridge, Massachusetts 02139, USA*

[2]*John A. Paulson School of Engineering and Applied Sciences, Harvard University, Cambridge, MA, USA*

[3]*Department of Physics, Harvard University, Cambridge, MA, USA*

[4]*Ernst Ruska-Centre for Microscopy and Spectroscopy with Electrons and Peter Grünberg Institute, Forschungszentrum Jülich, 52425 Jülich, Germany.*

[5]*Institut für Theoretische Physik, Universität Bremen, P.O. Box 330 440, 28334 Bremen, Germany*

[6]*Department of Chemistry, Columbia University, New York 10027, United States*

[7]*Department of Inorganic Chemistry, University of Chemistry and Technology Prague, Technická 5, 166 28 Prague 6, Czech Republic*

(Dated: 2022-07-12)

CONTENTS

Merged crystal structure. 3

Electron beam-induced structural changes. 4

Voronoi cell intensity and strain image analysis. 6

Time evolution of nucleation kinetics. 12

Twinning defects. 16

Stacking fault defect. 17

Cr migration and self-healing. 18

CrSBr material characterization. 19

References 22

MERGED CRYSTAL STRUCTURE.

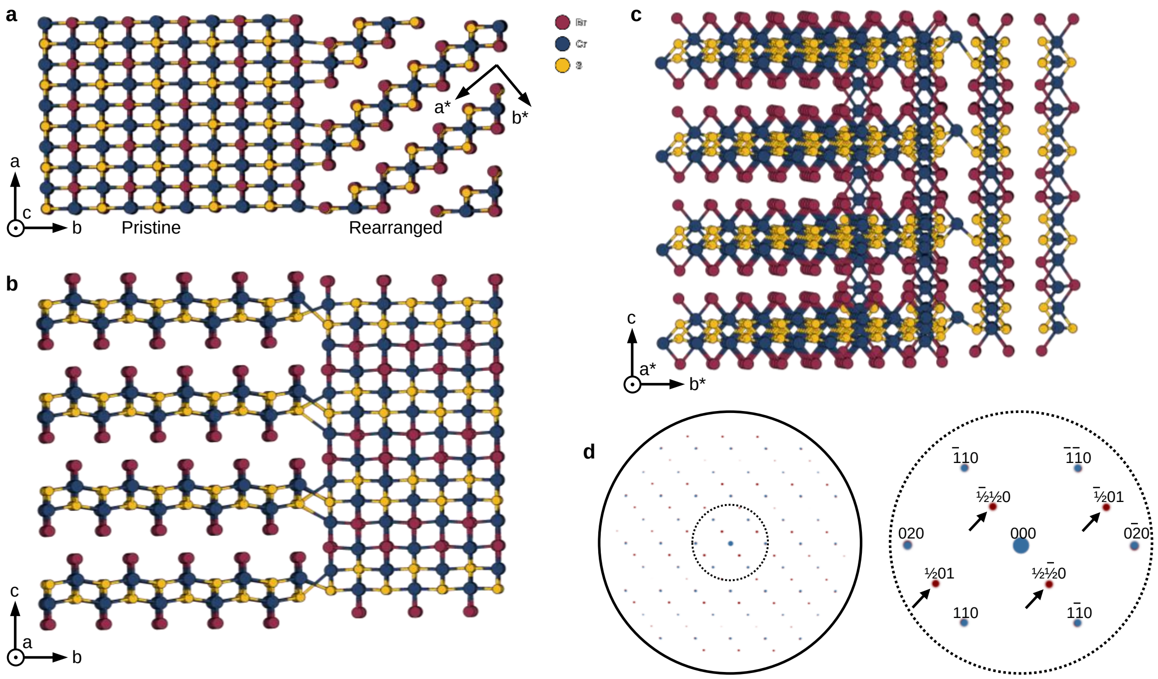

Supplementary Fig.| 1. Schematic diagrams of structures containing both pristine and rearranged CrSBr regions. a, Top-view. **b**, Side-view. **c**, View-along the rearranged layers. **d**, Simulated diffraction pattern. The arrowed spots originate from the rearranged structure.

ELECTRON BEAM-INDUCED STRUCTURAL CHANGES.

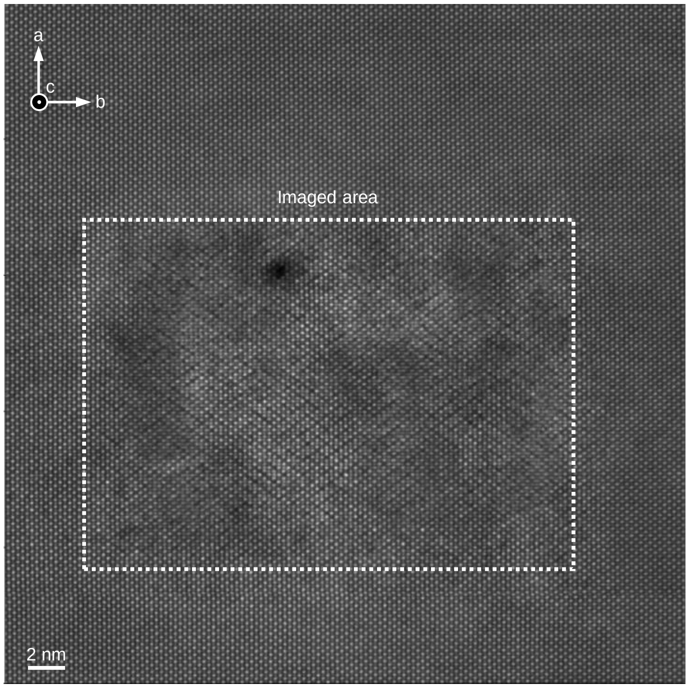

Supplementary Fig.| 2. **Electron beam-induced structural changes.** Low-magnification STEM-HAADF image at $200\,\mathrm{keV}$ of an electron beam dosed area.

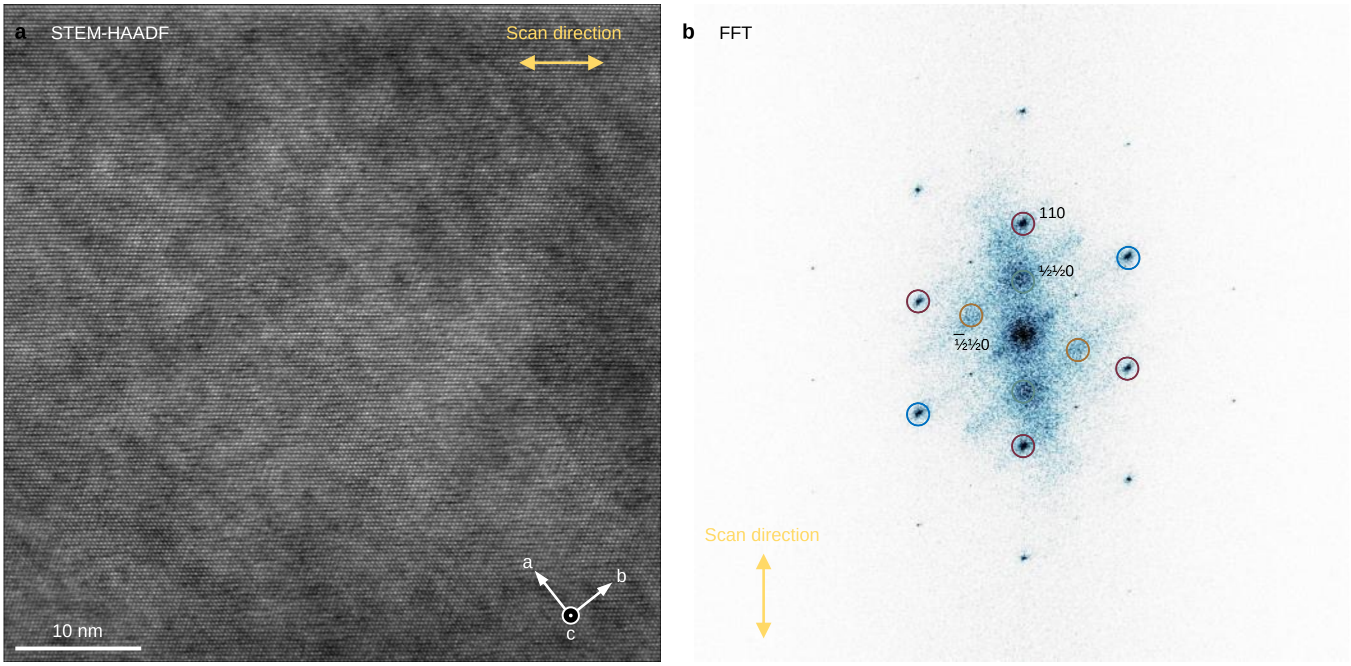

Supplementary Fig.| 3. **Electron beam-induced structural changes in a large area. a**, Low-magnification STEM-HAADF image at $200\,\mathrm{keV}$ of an electron beam dosed area. **b**, Corresponding FFT showing preferential rearrangement along the electron probe scan direction reflected by the higher intensity of the FFT peaks along the [110] direction.

VORONOI CELL INTENSITY AND STRAIN IMAGE ANALYSIS.

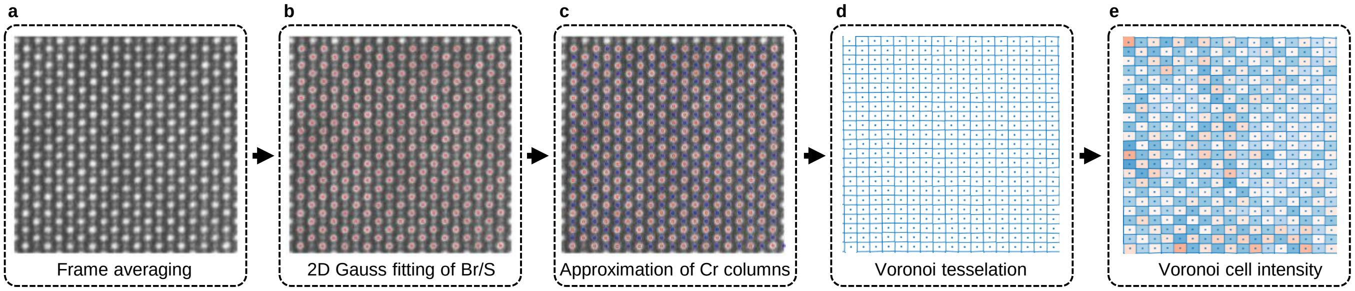

Supplementary Fig.| 4. Quantitative STEM-HAADF image analysis using Matlab. a, STEM-HAADF image of a $\sim 10\,\mathrm{nm}$ thin CrSBr flake after drift correction and frame averaging. In this example, four frames were averaged. **b**, Selective 2D Gauss fitting and indexing of the S/Br atom columns (red circles) using the atom column indexer. [1] **c**, Approximation of Cr atom column positions (blue circles) using four proximal S/Br positions that are obtained from the initial indexing through the 2D Gaussian fitting. **d**, Voronoi diagram of both the Cr and S/Br atomic columns obtained from a Voronoi tesselation. **e**, Corresponding Voronoi cell intensities of the S/Br (red) and Cr (blue) columns. The intensity of each Voronoi cell is obtained by masking out the polygon area from the initial image and by integrating the intensity of all pixels within the masked cell.

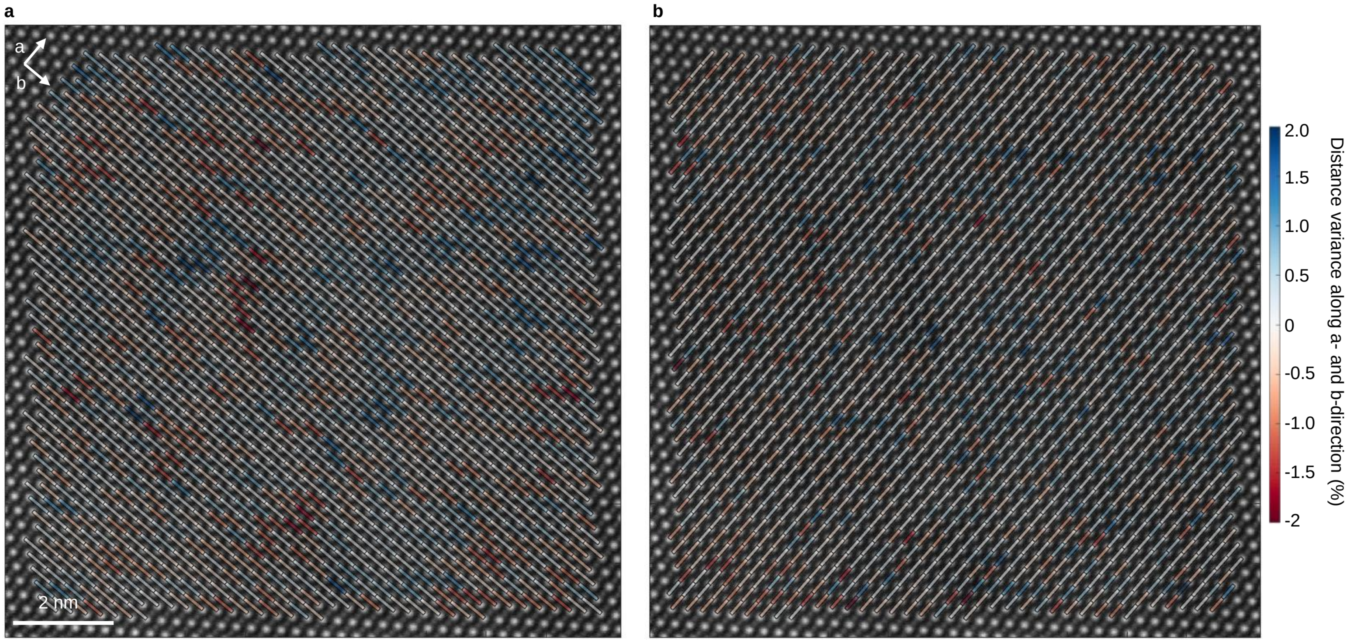

Supplementary Fig.| 5. **Local strain in CrSBr. a**, STEM-HAADF image of a pristine $\sim 10\,\mathrm{nm}$ thin CrSBr flake prior to electron beam dosing. **b**, **c**, Corresponding analysis of lattice distances along the a- and b-directions respectively, obtained from the indexed S/Br atom columns. The color of the bars plotted between the S/Br atom columns represent the deviation of the lattice parameter obtained from a normal distribution. The mean lattice parameters for the pristine image are $b = (477.82 \pm 3.03)\mathrm{pm}$ and $a = (346.46 \pm 2.14)\mathrm{pm}$ (see Supplementary Fig. 9)

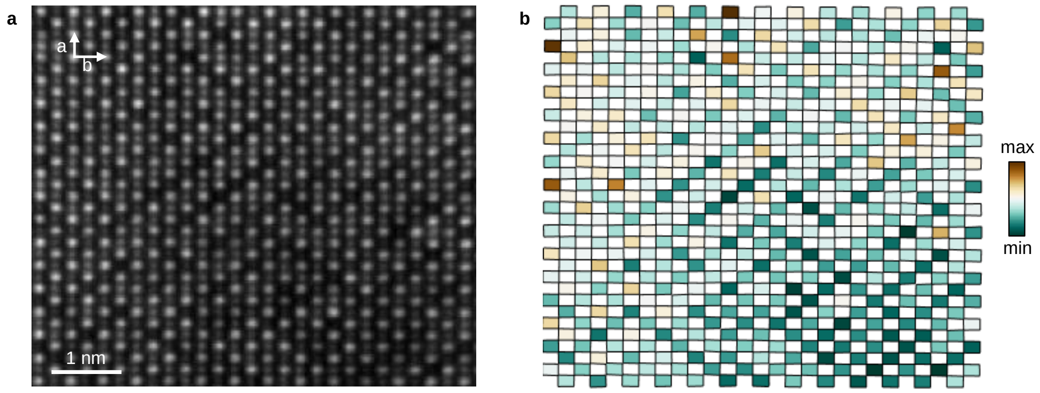

Supplementary Fig.| 6. Visualizing Cr rearrangement via Voronoi analysis. a STEM-HAADF image showing a maze-like pattern due to the rearrangement of Cr atoms between the columns along the diagonals ([110]) direction. **b** Voronoi diagram of the corresponding intensity of the Cr columns as obtained from the quantitative image analysis described in Supplementary Fig. 4.

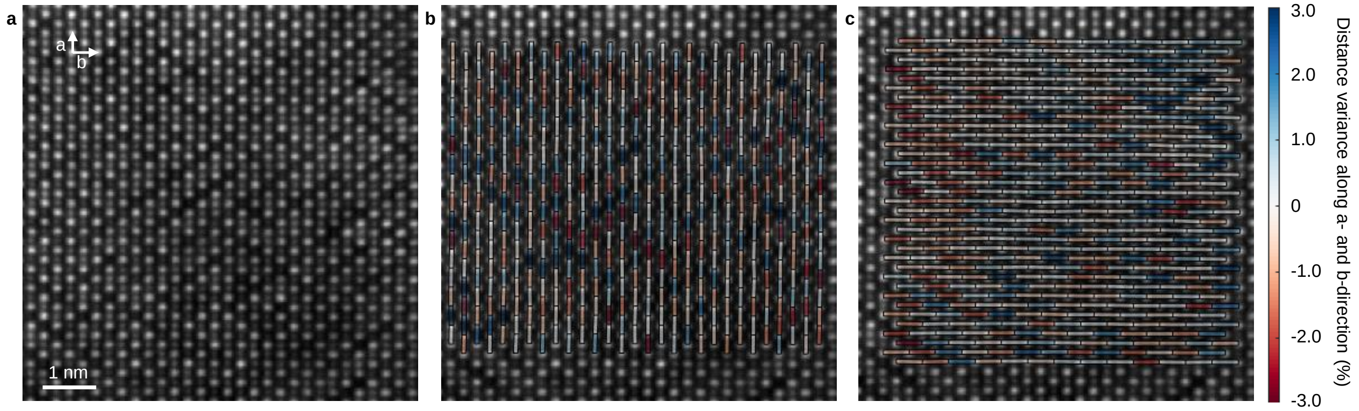

Supplementary Fig.| 7. Local strain of the rearranged CrSBr. a, STEM-HAADF image of an irradiated area revealing the maze-like structure of diagonal lines caused by the rearrangement of Cr atoms. **b**, **c**, Corresponding analysis of lattice distances along the a- and b-direction, respectively, obtained from the indexed S/Br atom columns. The mean lattice parameters for the pristine image are $b = (495.99 \pm 5.82)\mathrm{pm}$ and $a = (348.90 \pm 4.98)\mathrm{pm}$ (see Supplementary Fig. 4)

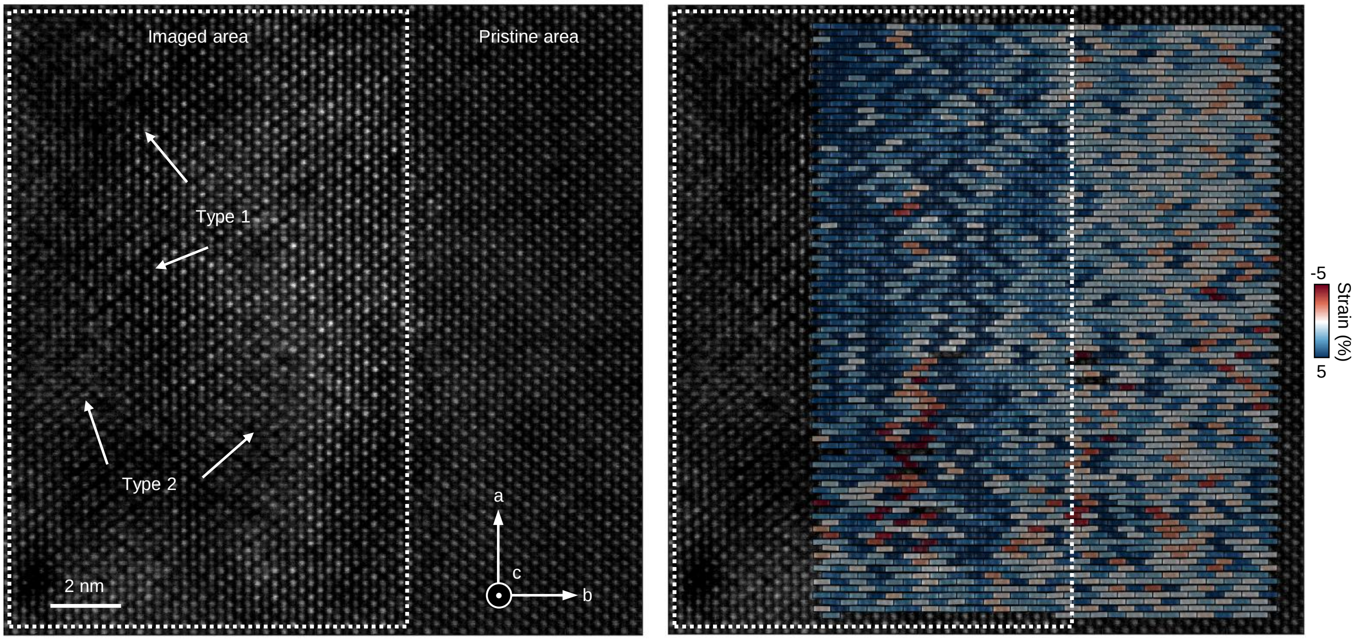

Supplementary Fig.| 8. Local strain analysis of an interface between electron-dosed and pristine CrSBr. Low-magnification STEM-HAADF image of an interface region between irradiated and pristine CrSBr and the corresponding atomistic strain analysis along the b-direction. The irradiated area shows type 1 and type 2 defects and increased strain along the b direction. The stacking fault defect (type) likely occurs due to reduce strain as suggested by negative relative strain values in those regions.

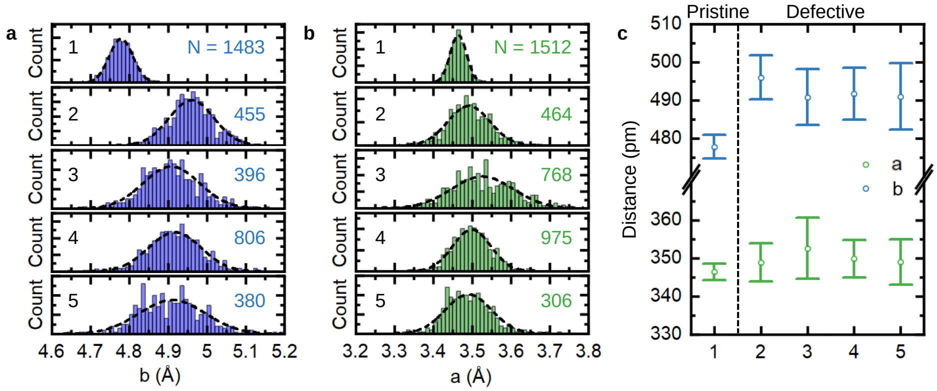

Supplementary Fig.| 9. **Statistics of lattice distances of pristine and defective CrSBr a**, Lattice parameters along the a-direction between the S/Br columns from quantitative STEM analysis. The histogram number 1 is from a pristine area (see Supplementary Fig. 5), while histograms number 2 - 5 are from several different areas after irradiation that reveal the 1D maze structure (see Supplementary Fig. 7) The number in the top right reflects the ensemble size (number of S/Br - S/Br distances). The dashed black line is a fitted normal distribution. **b**, Lattice parameters along the a-direction between the S/Br columns from quantitative STEM analysis. **c**, Comparison of a- and b-lattice distances obtained from fitted normal distributions from the histograms number 1 - 5 in **a** and **b**. **d**, The data suggest an elongation along the a-direction by about 3% while the b-direction stays constant within the statistical error.

TIME EVOLUTION OF NUCLEATION KINETICS.

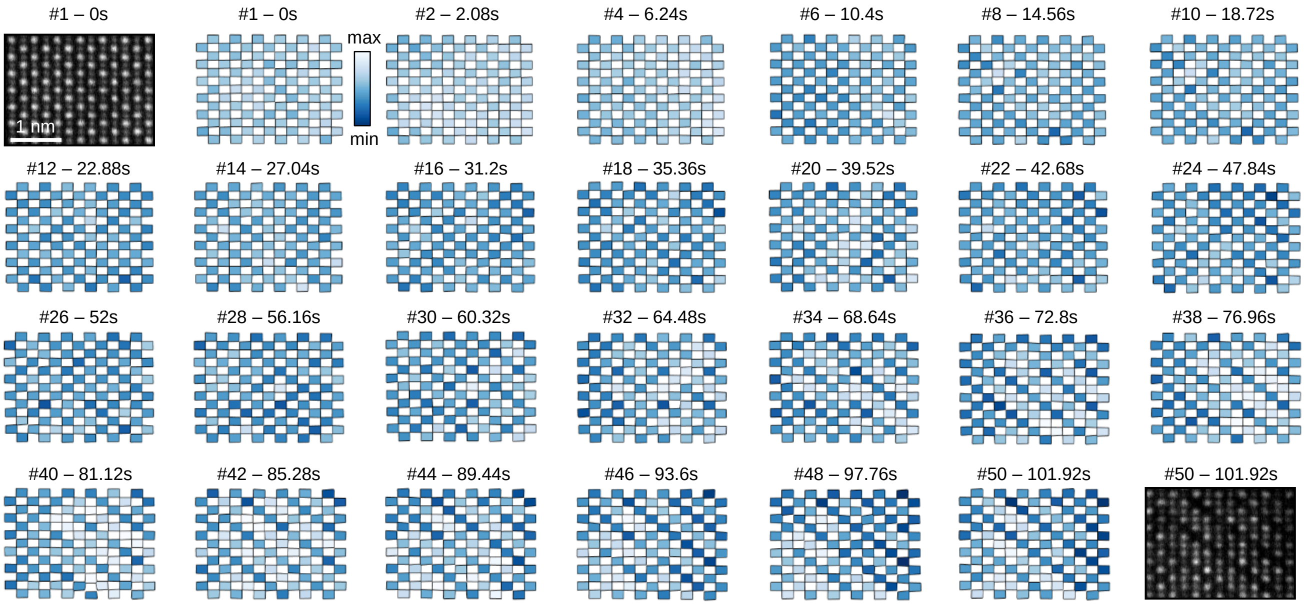

Supplementary Fig.| 10. Time dependent kinetics of the Cr rearrangement obtained from a Voronoi analysis of STEM-HAADF images. Voronoi diagrams of the Cr atom columns for selected frames from a sequence of STEM-HAADF images taken at a frame rate of $\sim 2\,\mathrm{Hz}$. In order to increase the signal-to-noise ratio for the data analysis, four frames were averaged resulting in a frame rate of $\sim 0.5\,\mathrm{Hz}$ for the analysis. The analysis described in Supplementary Fig. 4 was applied to each averaged frame.

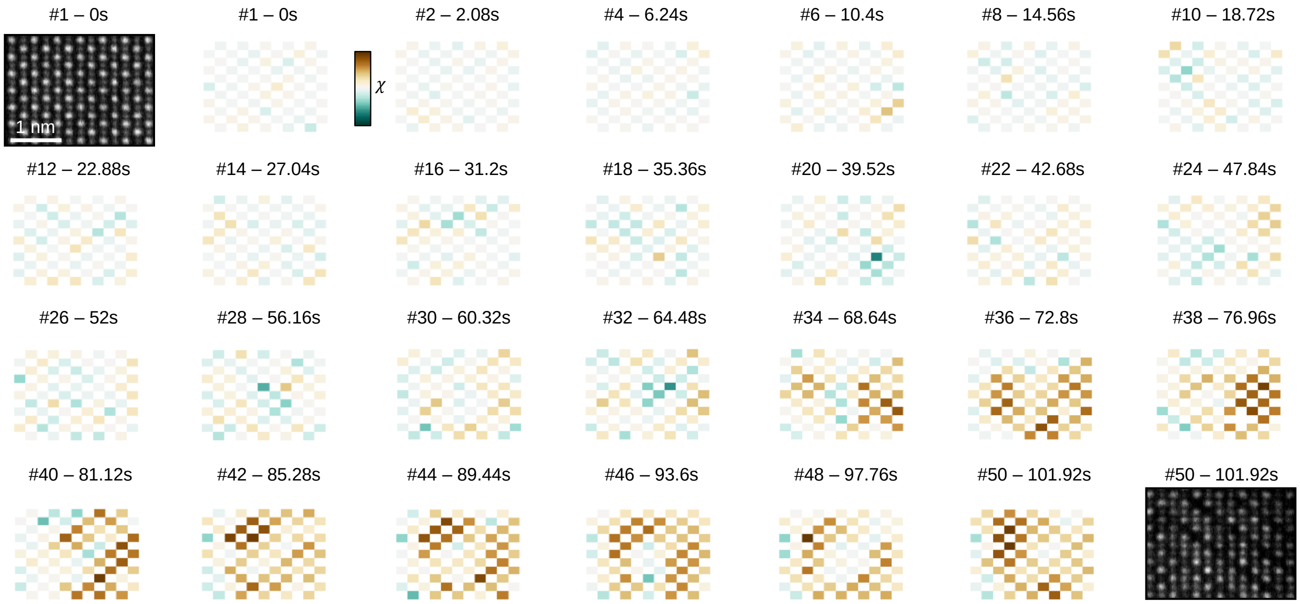

Supplementary Fig.| 11. **Time- and position-dependent initiation of the structural transformation.** The degree of ordering is calculated from nearest neighbor Cr atom column intensities (see Supplementary Fig. 10). The differences along diagonal bottom left to top right are defined as $\Delta_{15} = |I_1 - I_5|$ and $\Delta_{25} = |I_2 - I_5|$ while along diagonal top left to bottom right are defined as $\Delta_{35} = |I_3 - I_5|$ and $\Delta_{45} = |I_4 - I_5|$. A degree of ordering is then defined as $\chi = (\Delta_{15} + \Delta_{35}) - (\Delta_{25} + \Delta_{45})$.

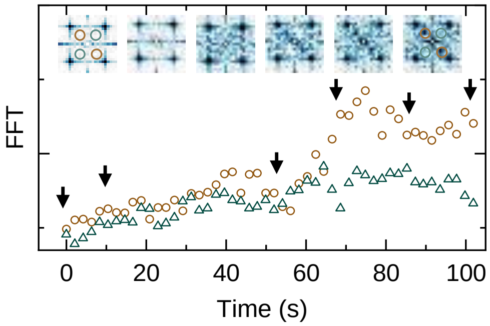

Supplementary Fig.| 12. **Time evolution of Cr rearrangement in Fourier space.** The intensity of the double periodicity spots in the FFT from a real-space image is integrated as a function of time. The spots are an indication of Cr rearrangement along the two different diagonals. Inset: Corresponding FFT images at $0\,\mathrm{s}$, $10.40\,\mathrm{s}$, $56.16\,\mathrm{s}$, $68.64\,\mathrm{s}$, $81.12\,\mathrm{s}$ and $101.92\,\mathrm{s}$. Similar to the real space analysis, there is a continuous increase for both orientations that changes into a preferred rearrangement along one Cr diagonal at $62\,\mathrm{s}$.

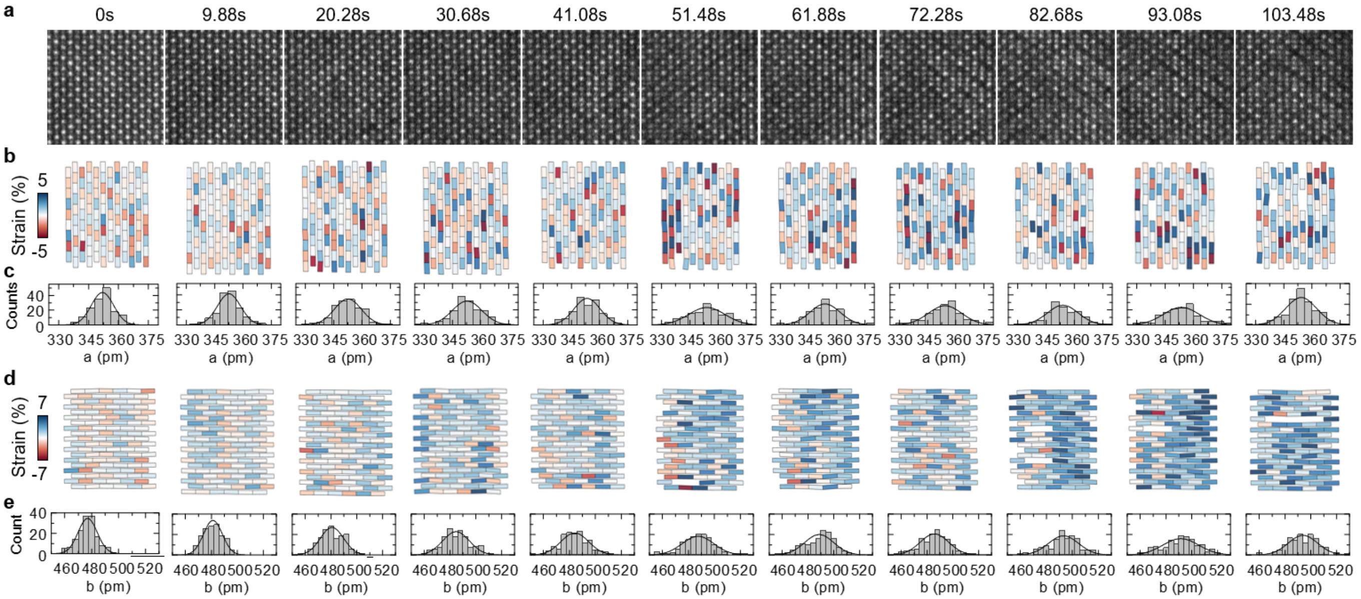

Supplementary Fig.| 13. **Time evolution of the 2D superlattice distortion kinetics and resulting net strain.** **a**, STEM-HAADF images of multilayer CrSBr taken at $200\,\mathrm{keV}$. **b**, Strain maps along the a-direction taking into account the positions of the S/Br atom columns. **c**, Corresponding a-distance histogram. **d**, Strain maps along the b-direction taking into account the positions of the S/Br atom columns. **e**, Corresponding b-distance histogram.

TWINNING DEFECTS.

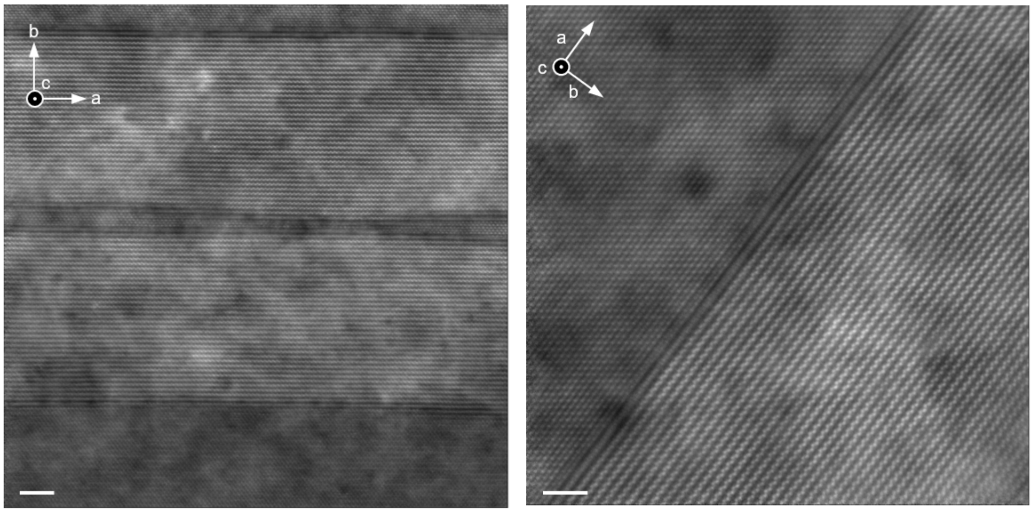

Supplementary Fig.| 14. **Twinning in CrSBr.** STEM-HAADF images of grain boundaries in CrSBr recorded at $60\,\mathrm{keV}$.The scale bar is $3\,\mathrm{nm}$ and $2\,\mathrm{nm}$.

STACKING FAULT DEFECT.

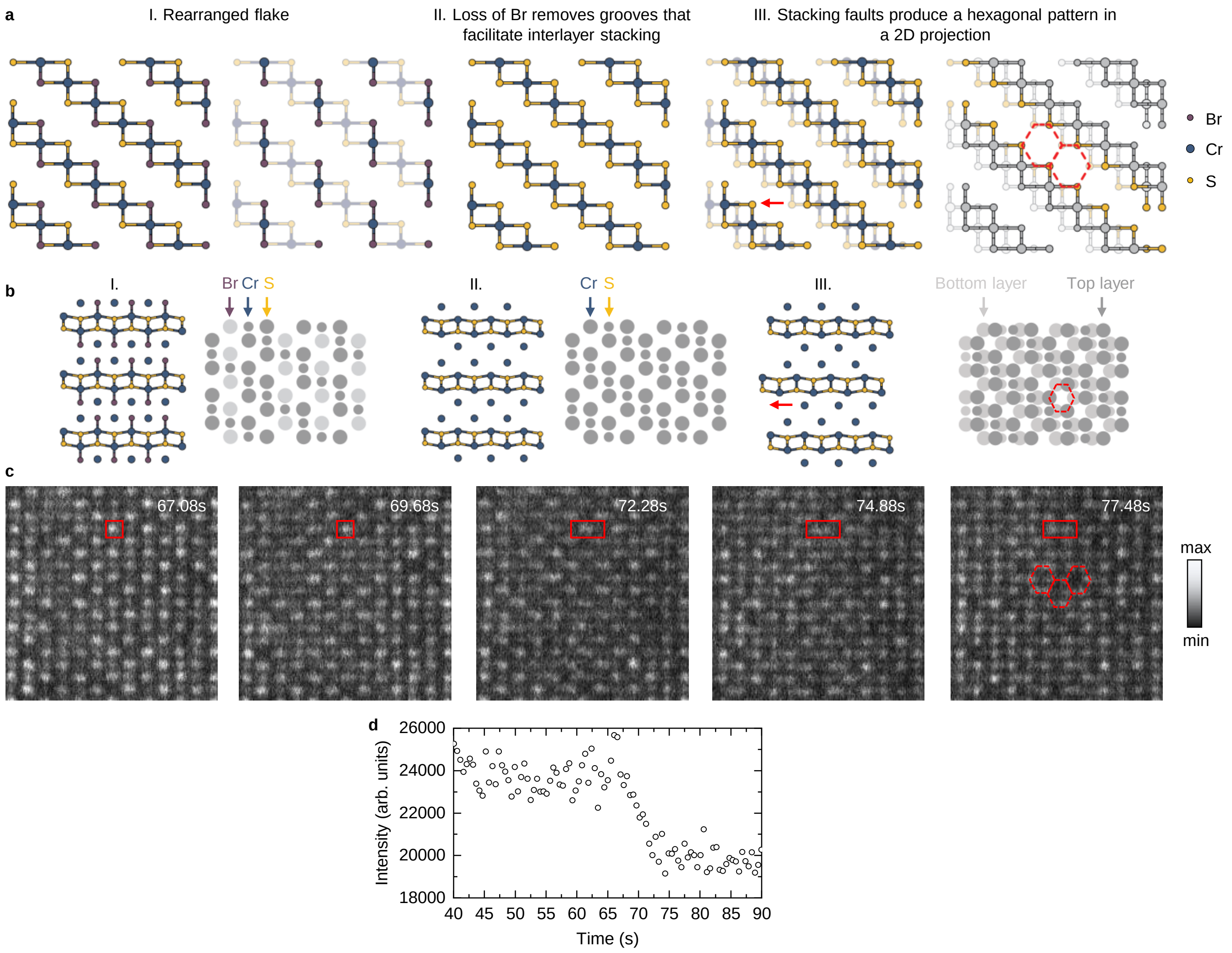

Supplementary Fig.| 15. **Type 2 irreversible stacking fault defect structure kinetics.** **a**, and **b**, Top view and side view schematic illustration of one possible crystallographic arrangement of atoms and individual atom planes under electron beam irradiation. Faded colors indicate multiple layers in which the bottom layers are shown with lower intensity. I. Type 1 rearranged structure. II. Beyond the regime where the type 1 rearranged structure forms, extended electron irradiation can result in the loss of atoms, presumably Br atoms forming Br_2 gas from proximal Br atoms which is released to the vacuum. III. Potential loss of Br atoms results in an increased mobility of individual layers consisting mostly of S and Cr that shift with respect to one another along the a-direction by $1.6\,\text{Å}$ showing a distorted hexagonal lattice pattern as a consequence. **c**, STEM-HAADF images for selected times taken from a sequence of data acquired while the type 2 defective structure is formed. A S/Br column is highlighted that appears to split in two columns as time elapses. The hexagonal pattern is highlighted by the red hexagons. **d**, Integrated atom column intensity as a function of time suggests knock-on damage and removal of material simultaneous with the formation of the type 2 defect structure.

CR MIGRATION AND SELF-HEALING.

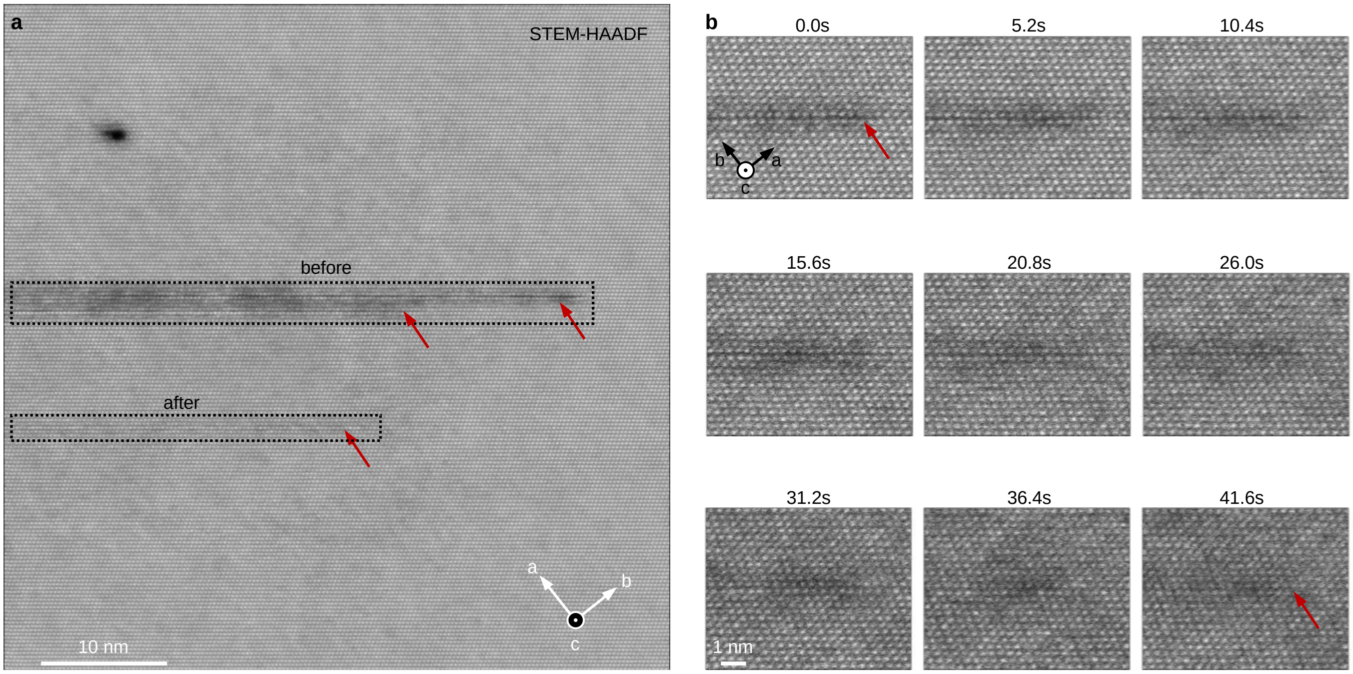

Supplementary Fig.| 16. **Self-healing of electron beam induced Cr vacancy line in CrSBr. a**, STEM-HAADF images Cr vacancy lines created by scanning the electron probe along the Cr diagonal. Two lines on the top are imaged before subsequent imaging and appear as dark lines. The line on the bottom was imaged for $40\,\mathrm{s}$ after generating the Cr vacancy line. The image is taken with an electron beam energy of $200\,\mathrm{keV}$. The Cr atoms migrate into the vdW gap due to the energy provided by the electrons resulting in a self-healing of the vacancy line when imaged. **b**, STEM-HAADF image series for an electron beam energy of $200\,\mathrm{keV}$ after generating a 1D Cr vacancy line.

CRSBR MATERIAL CHARACTERIZATION.

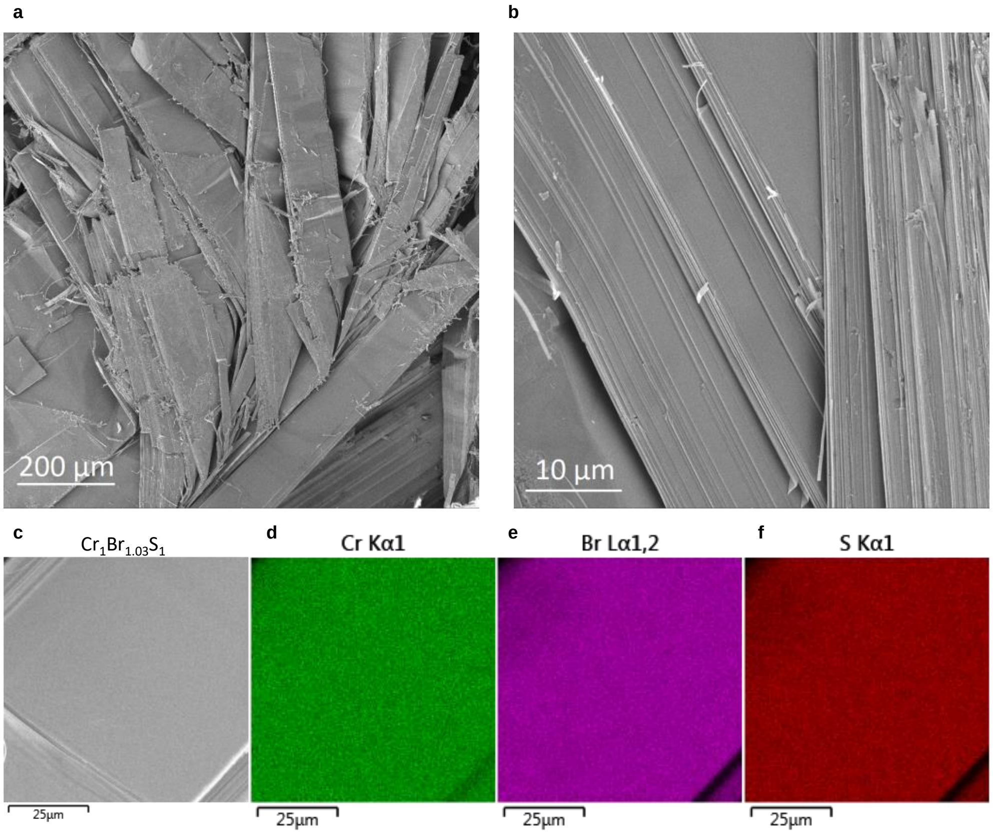

Supplementary Fig.| 17. **SEM and EDS of bulk CrSBr a** and **b**, SEM image of bulk CrSBr showing the layered nature and crystal anisotropy. The SEM images shows platelet character typical for van der Waals layerd materials. The EDS shows homogeneous distribution of Cr, Br and S. **c**, SEM image of bulk CrSBr. **d-f**, Corresponding EDS maps of the same region.

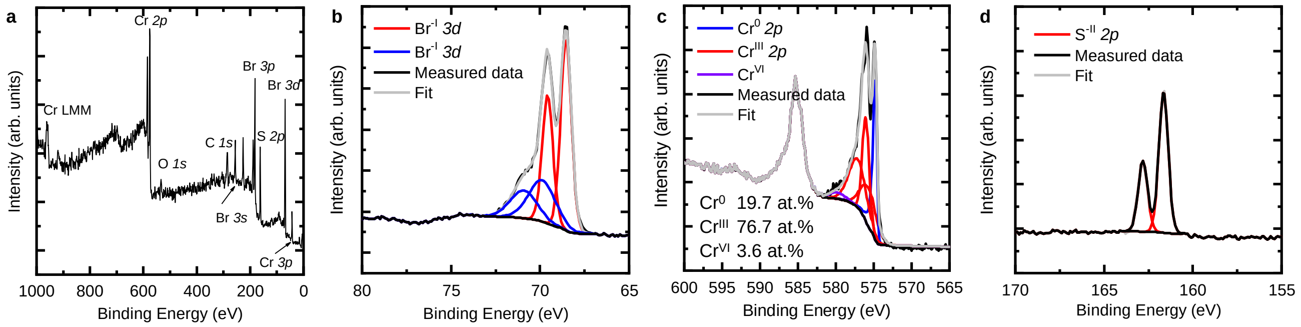

Supplementary Fig.| 18. **XPS of bulk CrSBr a-d**, High resolution XPS shows presence of Br^{-} and S^{2-} anions and chromium primarily in the $3+$ oxidation state.

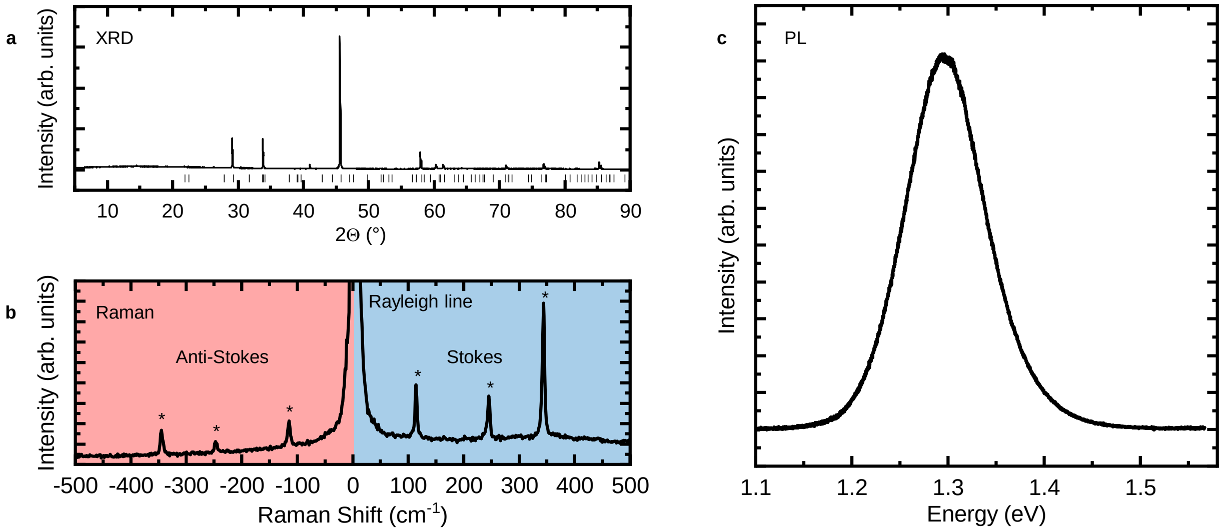

Supplementary Fig.| 19. **XRD, Raman and PL of bulk CrSBr a**, X-ray diffraction data corresponds to the pure single phase CrSBr with high preferential orientation due to layered structure. **b**, Room temperature Raman spectrum of multilayer CrSBr recorded with an excitation wavelength of $532\,\mathrm{nm}$ and a low-frequency filter set. **c**, Room temperature of multilayer CrSBr recorded with an excitation wavelength of $785\,\mathrm{nm}$.

* jpklein@mit.edu
† prineha@seas.harvard.edu
‡ fmross@mit.edu

[1] X. Sang, A. A. Oni, and J. M. LeBeau, Microscopy and Microanalysis **20**, 1764 (2014), URL https://doi.org/10.1017/s1431927614013506.
