# Supplementary material for: Control of structure and spin texture in the van der Waals layered magnet CrSBr
Source: Nat Commun. 2022 Sep 15;13:5420. doi: 10.1038/s41467-022-32737-8 (PMC9478124; doi:10.1038/s41467-022-32737-8)
Supplement: Supplementary file 2 — Supplementary Video legends [file 41467_2022_32737_MOESM2_ESM.docx]

**Supplementary Video 1**

Scanning transmission electron microscopy high-angle annular dark-field (STEM-HAADF) video of CrSBr multilayer showing the beam induced phase transformation of type 1. For acquisition an electron beam energy of 200 keV was used with a beam current of 75 pA. The pixel size is 1024x1024 with a pixel dwell time of 500 ns.

**Supplementary Video 2**

Scanning transmission electron microscopy high-angle annular dark-field (STEM-HAADF) video of CrSBr multilayer showing the beam induced layer stacking fault of type 2. For acquisition an electron beam energy of 200 keV was used with a beam current of 75 pA. The pixel size is 1024 x 1024 with a pixel dwell time of 500 ns.

**Supplementary Video 3**

Scanning transmission electron microscopy high-angle annular dark-field (STEM-HAADF) video of CrSBr multilayer showing a second example of a self-healing effect after cutting a 1D line into the CrSBr along the Cr lines highlighted by the red box. When imaged, neighboring Cr atoms get mobile under the beam and tend to move into the induced gap. For acquisition an electron beam energy of 200 keV was used with a beam current of 60 pA. The pixel size is 1024 x 1024 with a pixel dwell time of 500 ns.

**Supplementary Video 4**

Scanning transmission electron microscopy high-angle annular dark-field (STEM-HAADF) video of CrSBr multilayer showing a second example of a self-healing effect after cutting a 1D line into the CrSBr along the Cr lines highlighted by the red box. When imaged, neighboring Cr atoms get mobile under the beam and tend to move into the induced gap. For acquisition an electron beam energy of 200 keV was used with a beam current of 60 pA. The pixel size is 1024 x 1024 with a pixel dwell time of 500 ns.

**Supplementary Video 5**

Scanning transmission electron microscopy high-angle annular dark-field (STEM-HAADF) video of CrSBr multilayer showing the beam induced phase transformation of type 1. For acquisition an electron beam energy of 200 keV was used with a beam current of 105 pA. The pixel size is 1024 x 1024 with a pixel dwell time of 500 ns.
